# Cord blood metabolomics reveals gestational metabolic disorder associated with anti-thyroid peroxidase antibodies positivity

**DOI:** 10.1186/s12884-022-04564-8

**Published:** 2022-03-24

**Authors:** Lingna Han, Xin Yang, Wen Wang, Xueliang Yang, Lina Dong, Shumei Lin, Jianguo Li, Xiaojing Liu

**Affiliations:** 1 Department of Physiology, Changzhi Medical College, Changzhi, 046000 People’s Republic of China; 2 The First Affiliated Hospital of Xi’an JiaoTong University, 277 Yanta West Road, Xi’an, 710061 People’s Republic of China; 3 Institutes of Biomedical Sciences, Shanxi University, Taiyuan, 030006 People’s Republic of China; 4 Key Laboratory of Chemical Biology and Molecular Engineering of Ministry of Education, Shanxi University, 92 Wucheng Road, Taiyuan, 030006 People’s Republic of China

**Keywords:** Anti-thyroid peroxidase antibodies positivity, 1H-NMR metabolomics, Amino acid metabolism

## Abstract

**Background:**

Thyroid disease is one of the common endocrine disorders affecting the pregnant women, in which thyroid autoimmunity can alter the progress and the outcome of pregnancy. Women with euthyroid status but anti-thyroid peroxidase (anti-TPO) antibodies positivity before pregnancy are prone to subclinical gestational hypothyroidism. However, the connections between anti-TPO antibodies positivity and gestational hypothyroidism remain largely unknown. The aim of the present study is to investigate the differences of fetal metabolic profile at birth according to maternal anti-TPO status.

**Methods:**

We performed 1H-NMR metabolomics on cord blood of a nested case control cohort of 22 pregnant women with matched thyroid hormone levels and demographic data, including 11 women with euthyroid status but anti-thyroid antibodies positivity (into the anti-TPO antibodies positivity group) and 11 matched women as controls with euthyroid status and negative anti-thyroid antibodies (into the control group).

**Results:**

Distinct metabolic profiles were observed between the anti-TPO antibody positivity group and the nested control group, from which a total of 10 metabolites with between-group altered abundances were structurally identified. Five out of the 10 metabolites were up-regulated in the anti-TPO antibodies positivity group, including D-Glucose, L-Glutamine, 3-Hydroxybutyric acid, Myo-Inositol, Creatinine. The other 5 metabolites were down-regulated in the anti-TPO antibodies positivity group, including L-Leucine, L-Lysine, L-Glutamic acid, L-Tyrosine, and L-Phenylalanine. All the 10 metabolites have been previously reported to be correlated with hypothyroidism. Metabolite set enrichment analysis and pathway analysis suggested that amino acid metabolism pathways (especially the phenylalanine metabolism) were associated with anti-TPO antibodies positivity.

**Conclusion:**

The results of this study suggested that fetal metabolic disorder is correlated with anti-TPO antibodies positivity, representing by abundance alteration of hypothyroidism associated metabolites and the related disturbance of amino acid metabolism pathways.

**Supplementary Information:**

The online version contains supplementary material available at 10.1186/s12884-022-04564-8.

## Introduction

Gestational hypothyroidism (GH), as a common endocrine disorder in pregnant women, exerts a wide range of adverse maternal and fetal outcomes, including postpartum hemorrhage, miscarriage, low birthweight, preterm birth, respiratory distress, and risk for infectious morbidity of the offspring [1–6]. While a lot of attention has been focused on the routine screening, early confirmation of diagnosis and prompt treatment, the pathogenesis of GH remains largely undetermined. It is hypothesized that GH is developed due to thyroid regulatory mechanisms during pregnancy, inducing multiple pathophysiological insults in the hypothalamus–pituitary–thyroid axis [7]. 

Maternal factors are associated with gestational hypothyroidism, including maternal age, gestational diabetes mellitus, gestational age at antenatal visit, past obstetric history of miscarriages, and hypertension [2]. Anti-thyroid peroxidase (anti-TPO) antibodies positivity is one of the risk factors of GH, which is associated with higher TSH levels and higher risk of subclinical hypothyroidism [8]. Women with anti-TPO antibodies positivity before pregnancy are prone to develop GH [9]. It is believed that anti-thyroid antibodies positivity represents a generalized autoimmune imbalance that may be responsible for elevated complications despite the euthyroid status [10].

Metabolic syndrome is one of the representatives of increased complications in pregnant women with euthyroid status [11]. Metabolic syndrome is resulted from abnormal chemical reactions in the body, which contributes to numerous gestational diseases, including gestational diabetes mellitus [12], gestational hypertension [13], gestational hypothyroidism [14], etc. Metabolic disorder also exerts direct adverse effects to the progress and outcome of pregnancy [15]. Thus, more attention should be paid on the metabolic disorder induced by anti-thyroid antibodies positivity in the pregnant women with euthyroid status.

Metabolic adaptations represent a crucial part of pregnancy, providing sufficient energy to the mother to meet the demands of pregnancy. Aberrant metabolic adaptations result in metabolic disorders. Metabolic disorders during pregnancy are risk factors of pregnancy complications and postpartum cardiovascular diseases [16–18]. The maternal and fetal compartments are different and are separated by the placenta interface. The altered fetal metabolic status can drive various consequences, which is critical in gestational and postpartum health of mother and the fetus/offspring.

Many clinical factors could influence the metabolic status in pregnant women, including age [19], BMI (body mass index) [20], levels of TSH (thyroid stimulating hormone) [21, 22], FT3 (free triiodothyronine) or FT4 (free thyroxine) [23]. Complicated clinical manifestations confound the investigations in figuring out the role of anti-thyroid antibodies positivity [24, 25]. To investigate the differences of fetal metabolic profile at birth according to the status of maternal anti-TPO antibodies, we performed a 1H-NMR metabolic profiling of cord blood from a nested case control cohort with matched clinical status in the present study.

## Materials and methods

### Study design

We retrospectively assessed a prospectively collected cohort of pregnant women in the First Affiliated Hospital of Xi'an JiaoTong University, (Xi'an, PR China) between April and September of 2018. A cohort of 22 pregnant women with singleton pregnancy were enrolled, including 11 women with euthyroid status but anti-thyroid antibodies positivity (into the anti-TPO antibodies positivity group) and 11 matched women as controls with euthyroid status and negative anti-thyroid antibodies (into the control group). As an exploratory study, the sample size was determined based on available resources and logistic limitations (it is difficult to obtain a larger cohort of nested matched case & control for the investigation of anti-TPO antibodies positivity).

Written informed consent has been obtained from all the human participants. According to the 2017 Guidelines of the American Thyroid Association for the Diagnosis and Management of Thyroid Disease During Pregnancy and Postpartum [26], the threshold for anti-TPO antibodies positivity was set at 34 IU/ml, and euthyroid status was defined as normal levels of thyroid hormones (0.27 μIU/ml < TSH < 4.2 μIU/ml, 3.1 pmol/L < FT3 < 6.8 pmol/L, and 12 pmol/L < FT4 < 22 pmol/L) The controls were matched with the women with anti-TPO antibodies positivity by age, BMI, fasting blood glucose, TSH, FT3, FT4, gestational weeks of delivery, fetus number, birthweight and gender of the newborn (Table 1). Pregnant women with any known pre-existing thyroid dysfunction, impaired fasting glucose, abnormal liver, or kidney function were excluded. All the enrolled pregnant women exhibited no symptom of gestational diabetes or gestational hypertension. The study was performed in accordance with the Declaration of Helsinki, following the STROBE guidelines for reporting of observational studies [27], and had been approved by the Ethics Committee of the First Affiliated Hospital of Xi'an JiaoTong University.

**Table 1:** Demographic data and clinical chemistry test results for the enrolled pregnant women and the newborns in the present study

Item	Control group (mean ± s.d.)	Anti-TPO antibodies positivity group (mean ± s.d.)	*p*-value
Age (year)	29.42 ± 2.17	30.09 ± 3.08	0.26
BMI of the pregnant women (Kg/m^2^)	26.29 ± 2.74	26.27 ± 3.48	0.76
Fetus number (singleton/multiple pregnancies)	6/5	9/2	0.18 (χ^2^ = 2.93)^*^
gestational weeks of delivery	38.85 ± 0.95	38.91 ± 0.83	0.69
TSH (μIU/ml)	2.20 ± 0.37	2.28 ± 0.32	0.91
FT3 (pmol/L)	4.78 ± 0.47	4.85 ± 0.48	0.48
FT4 (pmol/L)	15.59 ± 2.10	16.47 ± 2.57	0.30
anti-TPO antibodies (IU/ml)	15.11 ± 4.16	273.70 ± 168.50	0.00047
FBG in the 11th gestational week (mmol/L)	4.65 ± 0.19	5.18 ± 0.69	0.06
FBG at delivery (mmol/L)	4.44 ± 0.32	4.91 ± 0.60	0.09
Gender of the newborns (Male/Female)	5/6	6/5	0.39 (χ^2^ = 0.78)^#^
Birthweight of the newborns (Kg) (Q1, Q2, Q3)^a^	3.32 ± 0.35 (3.17, 3.38, 3.51)	3.14 ± 0.21 (3.02, 3.09, 3.27)	0.52

*BMI* body mass index, *TSH* thyroid stimulating hormone, *FT3* free triiodothyronine, *FT4* free thyroxine, *FBG* fasting blood glucose

^a^The distribution of newborn weight was expressed by the first Quartile (Q1), the second Quartile (Q2), and the third Quartile (Q3)

*, # The differences of fetus number* and newborn gender# between the case and the control group were tested by the chi-square test, respectively

### Demographic data collection and clinical chemistry tests

Demographic data of the enrolled pregnant women were obtained by an interview during the 11^th^-13^th^ weeks of gestation, including age, nationality, body weight, blood pressure and disease history. Fasting blood was collected simultaneously for the detection of TSH, FT3 and FT4. Cord blood was collected during birth-giving. Heparinized plasma was obtained by centrifuge of the collected blood samples at 3000 rpm for 5 min, aliquoted and stored in a -86℃ deep freezer. Thyroid hormones and anti-TPO antibodies were measured with the fasting plasma collected during the 11^th^-13^th^ weeks of gestation by a Cobas 6000 analyzer (Roche Diagnostics, Basil Switzerland) utilizing the electrochemiluminescence (ECL) technology. The levels of anti-TPO antibodies were determined by using the Roche Diagnostics COBAS Elecsys Anti‑TPO performed with a Cobas 6000 analyzer according to the manufacture’s instructions. Fasting blood glucose was measured by using an AU5800 Automatic Biochemical Analysis System (Beckman Coulter, CA, USA).

### 1H-NMR based metabolic profiling

Cord blood sample preparation for 1H-NMR spectral profiling was performed according to a previously reported procedure [28]. Briefly, a volume of 450 μl sample was mixed with 900 μl methanol (HPLC grade), vortexed for 5 min and then centrifuged at 4℃, 15, 000 rpm for 20 min for protein precipitation. The supernatant was dried completely by a vacuum dryer (Concentrator plus, Eppendorf, Germany), and reconstituted in 600 μl phosphate buffer (0.2 M NaH_2_PO_4_/Na_2_HPO_4_ in D_2_O, containing 10 mM TSP as internal standard, pD = 7.4). The reconstituted sample was then centrifuged at 4℃, 15, 000 rpm for 20 min to eliminate any precipitate. A volume of 550 μl of the supernatant was transferred into a 5 mm NMR tube for NMR spectral profiling. A Bruker 600 MHz AVANCE IIII NMR spectrometer (Bruker BioSpin, Germany) was applied for NMR spectral profiling, with the spectrum acquired by using the CPMG (Carr-Purcell-Merboom-Gill) pulse sequences. The parameters for CPMG were as follows: spectral width, 12,019.2 Hz; pulse width, 14 μs; spectral size, 65,536 points; relaxation delay, 1.0 s; number of scans, 64. MestReNova (v8.0.1) was applied for spectral data processing. The baseline and phase were corrected manually and the chemical shift of the internal standard TSP was calibrated at 0.00 ppm. The spectra region of δ 0.08 to δ 9.05 was segmented at 0.01 ppm width into NMR features with exclusion of the region of residual water (δ 4.63–δ 5.02).

Between-group differential NMR features were determined by the following criteria: Importance for the Projection (VIP) ≥ 1 in the OPLS-DA model; false discovery rate (fdr)-adjusted *P* < 0.05 in between-group t test of the relative peak area of the NMR features; the absolute value of p(corr) in S-Plot analysis by SIMCA-P greater than 0.40 (determined by inquiring the critical value table of Correlation Coefficient Test). Metabolites were identified by searching the HMDB database (https://www.hmdb.ca) with the chemical shift and the coupling constant of the NMR features. Metabolic pathway prediction was performed by the implemented modules (the metabolite set enrichment analysis module and the pathway analysis module) in MetaboAnalyst [29] (v4.0, https://www.metaboanalyst.ca).

### Multivariate statistical analysis

SIMCA-P (v14.1) was applied for multivariate statistical analysis of the metabolomic data. Principal component analysis (PCA) was performed for an overview of the metabolomic data and exclusion of any sample with abnormal value (determined by the Hotelling’s T2 range). Orthogonal projection to latent structures discrimination analysis (OPLS-DA) was applied to investigate the between-group variations by incorporating the classification information. A 200-cycle permutation test was performed to assess the fitness of the selected OPLS-DA model. The parameters multiple correlation coefficient (R2) and the cross-validated R2 (Q2) were applied for evaluating the fitting validity and the predictive ability of the selected OPLS-DA model, respectively. Data of metabolic profiling, clinical and demographic parameters were expressed as mean ± s.d. Mann–Whitney U test implemented in GraphPad Prism (v9.0.0) was applied for between-group statistical analysis unless otherwise specified. False discovery rate (FDR) was used as the post-hoc test method for multiple comparisons of the metabolic profiling data. *p* < 0.05 was considered statistically significant.

## Results

### Data pre-processing and quality control of the 1H-NMR metabolomics

Missing values of the metabolomic data were first detected by a data integrity check and were excluded with an in-house kept KNN (K-nearest neighbors) algorithm before subsequent analysis. The metabolomic data were then subjected to normalization procedures, including normalization by sum, log transformation, and Pareto scaling. A better distribution after normalization was observed (Figure S1). Quality control (QC) samples were applied to monitor the reproducibility of sample preparation and the robustness of instrument analysis. QC was performed by PCA on the tested samples and the QC samples (derived from a pool of aliquot from each sample). All the QC samples were clustered together in the sample space of PCA (Figure S2), suggesting good reproducibility and robustness of the metabolomic profiling.

### Anti-thyroid antibodies positivity associated metabolic disorder in cord blood

**Fig. 1:**
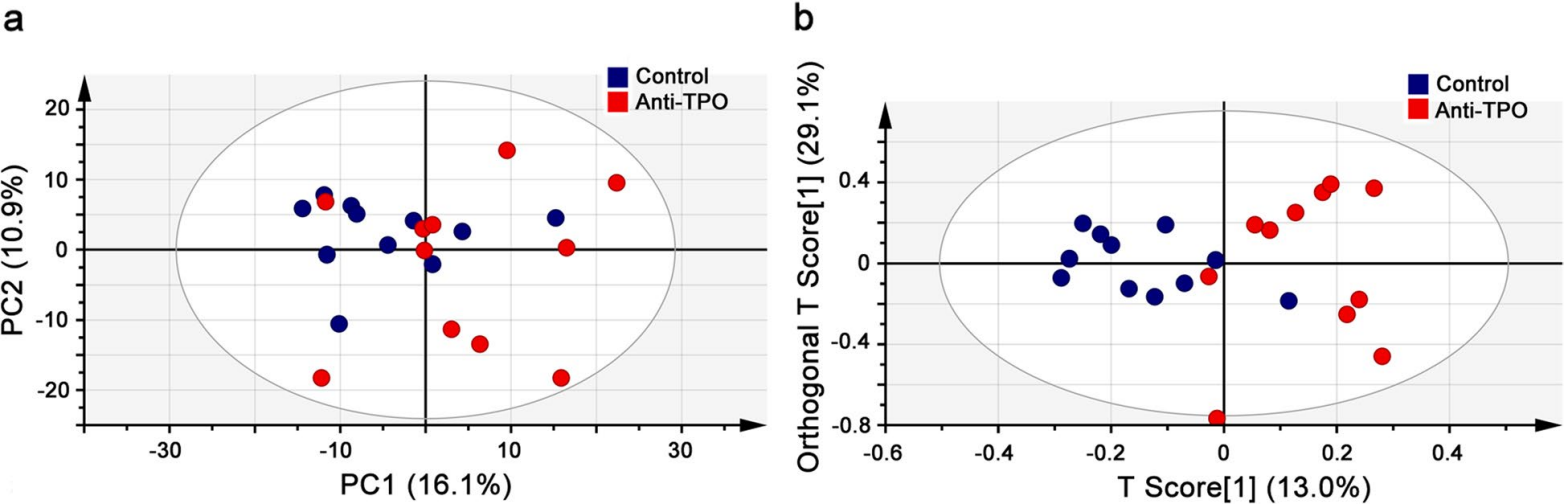
PCA and OPLS-DA on the cord blood metabolome between pregnant women with anti-TPO antibodies positivity and the nested control. **a** PCA score plot based on the first two principle components (PCs), which explain 27% of the total variations (16.1% and 10.9% for PC1 and PC 2, respectively). **b** OPLS-DA score plot of the cord blood metabolome. The horizontal axis represents the predicted score of the first component, which explained 13% of the between-group variations. The vertical axis represents the orthogonal principle components score, which explained 29.1% of the within-group variations. The ellipse represents the 95% confidence interval using Hotelling’s T^2^ statistics. PCA: Principle Component Analysis. OPLS-DA: Orthogonal Partial Least Squares- Discriminant Analysis. anti-TPO: Anti-thyroid peroxidase

We first performed PCA on the cord blood metabolome to evaluate the natural separation and to exclude any outliers (Figure S3a). Two principle components (PCs) were automatically selected, which explained 27% of the total variations (16.1 and 10.9% for PC1 and PC2, respectively). The metabolome of the control group clustered and showed a separation trend to that of the anti-TPO antibodies positivity group (Fig. 1a), indicating that metabolic disorders associated with anti-TPO antibodies positivity occurred.

To better discriminate the cord blood metabolome between the anti-TPO antibodies positivity group and the control group, we carried out a supervised OPLS-DA, incorporating the known sample classification information (Fig. 1b). The best-fitted OPLS-DA model was automatically selected, and then evaluated by a cross-validation of all available models using a 200-cycle permutation test (Figure S3b). The selected OPLS-DA model exhibited acceptable fit goodness (R2 = 0.54) and prediction ability (Q2 = -0.50). A clear separation was observed between the anti-TPO antibodies positivity group and the control group (Fig. 1b), confirming that fetal metabolic changes occurred in pregnant women with anti-thyroid antibodies positivity.

### Identification of the altered cord blood metabolites associated with anti-TPO antibodies positivity

**Fig. 2:**
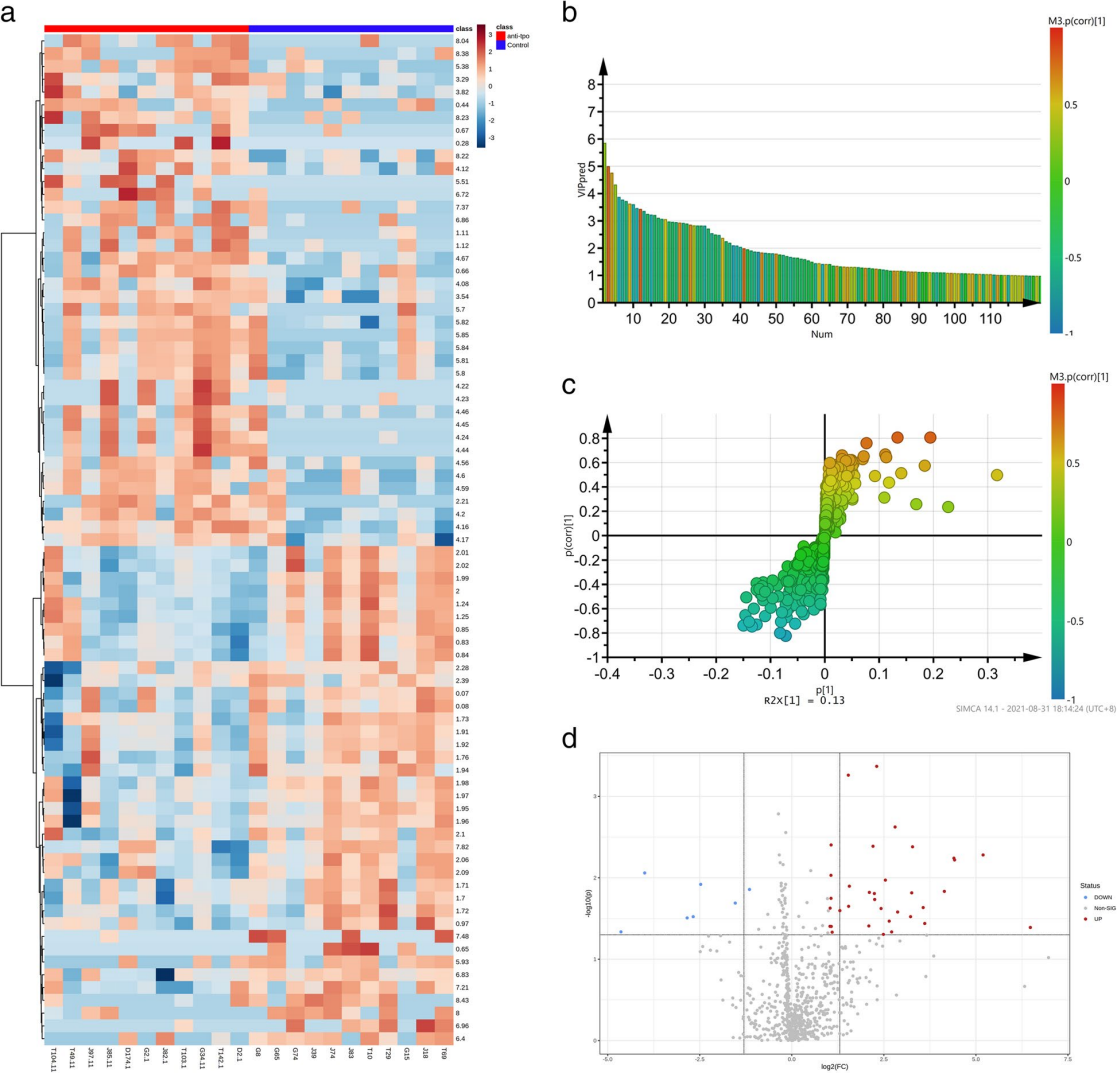
Identification of the cord blood metabolites with altered abundance associated with anti-TPO antibodies positivity. **a** Hierarchical clustering of the top 79 1H-NMR features with altered abundances in the anti-TPO antibodies positivity group. Euclidean distance and the Ward method were used to generate clustering tree. Abundances of the 1H-NMR features were displayed as colors ranging from red to blue as shown in the color bar (red, higher abundance, blue; lower abundance). **b** Variable Importance (VIP) plot of the top 39 1H-NMR features with VIP > 1. The horizontal axis represents the 1H-NMR features; the vertical axis represents the values of the corresponding 1H-NMR features. The columns were colored according to the values of spearman rank correlation coefficient as shown in the color bar. The detailed VIP values are listed in Table S1. **c** S-Plot constructed from the OPLS-DA model shown in Fig. 1b, showing the covariance *p* [1] against the correlation coefficient *p*(corr) [1] of the 1H-NMR features with altered abundances in the Anti-TPO antibodies positivity group. The solid cycles were colored according to the values of spearman rank correlation coefficient as shown in the color bar. The identified metabolites with altered abundance in the anti-TPO antibodies group were labeled. The corresponding data are listed in Table S2. **d** Volcano plot of the 1H-NMR features with between-group altered abundances. The horizontal axis displays the log twofold change (FC) value, and the vertical axis represents the value of log 10 FDR-adjusted *p* value. The thresholds were set at FC ≥ 2, and FDR-adjusted *P* < 0.05. The blue and red circles denote the down- and up-regulated 1H-NMR features in the anti-TPO antibodies positivity group, respectively. The grey circles denote the 1H-NMR features without marked differences. The corresponding data are listed in Table S3

A total of 741 features were obtained from the 1H-NMR spectrum by binning in 0.01 ppm increments. Seventy nine out of the 741 features with altered between-group abundances (*P* < 0.05 in a Mann–Whitney U test) were divided into two clusters (Fig. 2a). One cluster of 40 features were enriched in the anti-TPO antibodies positivity group (the red rectangular box in Fig. 2a), while the other cluster of 39 features were enriched in the control group (the blue rectangular box in Fig. 2a). Thirty two out of the 79 features with between-group differential abundances were further selected by multiple testing adjustment according to the following criteria: VIP (Variable Importance for the Projection) > 1 (Fig. 2b, Table S1); |p(corr)| (the absolute value of spearman rank correlation coefficient) > 0.40 (Fig. 2c, Table S2); between-group fold change (FC) > 2 and *adjusted P* < 0.05 in Mann–Whitney U test (Fig. 2d, Table S3).

**Fig. 3:**
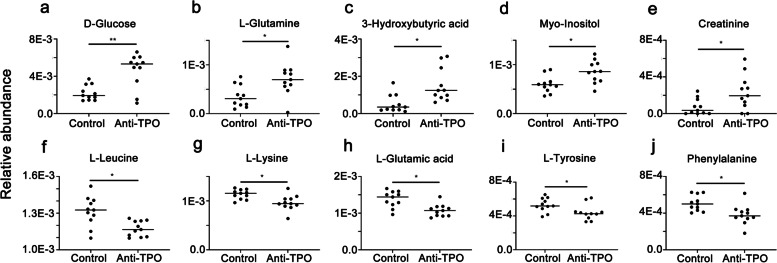
Relative abundance of the altered cord blood metabolites between pregnant women with anti-TPO antibodies positivity and the nested control. **a** D-Glucose; **b** L-Glutamine; **c** 3-Hydroxybutyric acid; **d** Myo-Inositol; **e** Creatinine; **f** L-Leucine; **g** L-Lysine; **h** L-Glutamic acid; **i** L-Tyrosine; **j** L-Phenylalanine. The relative abundance of a metabolite was defined as the corresponding peak area divided by the total peak area of one cord blood sample. The between-group statistic significance was calculated with Mann–Whitney test. * *p* < 0.05; ** *p* < 0.01

Ten metabolites were structurally identified from the 1H-NMR features with altered between-group abundances (Table 2), including 60% (6/10) amino acids (L-Leucine, L-Lysine, L-Glutamic acid, L-Glutamine, L-Tyrosine, and Phenylalanine), 10% (1/10) monosaccharide (Glucose), 10% (1/10) liposoluble vitamin (Myo-Inositol), 10% (1/10) fatty acid derivate (3-Hydroxybutyric acid), and 10% (1/10) organic acid derivate (Creatine). The relative abundances of the identified metabolites demonstrated that 50% (5/10) metabolites (Fig. 3 a-e) were significantly up-regulated in the anti-TPO antibodies positivity group, including Glucose (*p* < 0.01), L-Glutamine (*p* < 0.05), 3- Hydroxybutyric acid (*p* < 0.05), Myo-Inositol (*p* < 0.05), and Creatinine (*p* < 0.05). And the other 50% (5/10) metabolites were significantly down-regulated (Fig. 3 f-j) in the anti-TPO antibodies positivity group, including L-Leucine, L-Lysine, L-Glutamic acid, L-Tyrosine, and L-Phenylalanine. These results suggested that anti-thyroid antibodies positivity was associated with metabolic disorder in cord blood, including metabolism of glucose, amino acids, and fatty acid derivate.

**Table 2:** Cord blood metabolites with altered abundances induced by anti-thyroid antibodies positivity

Metabolite	Chemical shift	anti-TPO/Control				
		**VIP**	**P(corr)**	**FC**	***p*-value**	**FDR-adjusted** ***p*-value**
L-Leucine	0.948t, 1.700 m, 3.722 m	1.01	-0.42	0.06	< 0.01	< 0.01
L-Lysine	1.46 m, 1.71 m, 1.89 m, 3.02t, 3.74t	1.35	-0.55	0.18	< 0.01	< 0.05
D-Glucose	3.233dd, 3.398 m, 3.458 m, 3.524dd, 3.728 m, 3.824 m, 3.889dd, 4.634d, 5.223d	5.51	0.63	2.09	< 0.01	< 0.01
Creatinine	3.03 s, 4.05 s	1.02	0.59	2.10	< 0.01	< 0.01
Myo-Inositol	3.268t, 3.524dd, 3.613t, 4.053t	1.17	0.53	4.61	< 0.01	< 0.01
3-Hydroxybutyric acid	1.204d, 2.314dd, 2.414dd, 4.160 m	3.44	0.81	2.05	< 0.01	< 0.05
L-Glutamic acid	2.040 m, 2.119 m, 2.341 m, 3.748dd	1.92	-0.76	0.14	< 0.05	< 0.05
L-Glutamine	2.125 m, 2.446 m, 3.766t	2.48	0.66	5.82	< 0.01	< 0.05
L-Tyrosine	3.024dd, 3.170dd, 3.921dd, 6.877 m, 7.170 m	1.49	-0.69	0.35	< 0.01	< 0.05
L-Phenylalanine	3.19 m, 3.98dd, 7.32d, 7.36 m, 7.42 m	1.46	-0.64	0.16	< 0.01	< 0.01

*VIP* Variable Importance in the projection, *FDR* false discovery rate, *p(corr)* spearman rank correlation coefficient calculated with the principle component 1 of the selected OPLS-DA model, *FC* Fold change, *s* single peak, *d* double peaks, *t* triplet peaks, *q* quarter peaks, *m* multiple peaks

### Anti-thyroid antibodies positivity was associated with disturbance in amino acid metabolic pathways

**Fig. 4:**
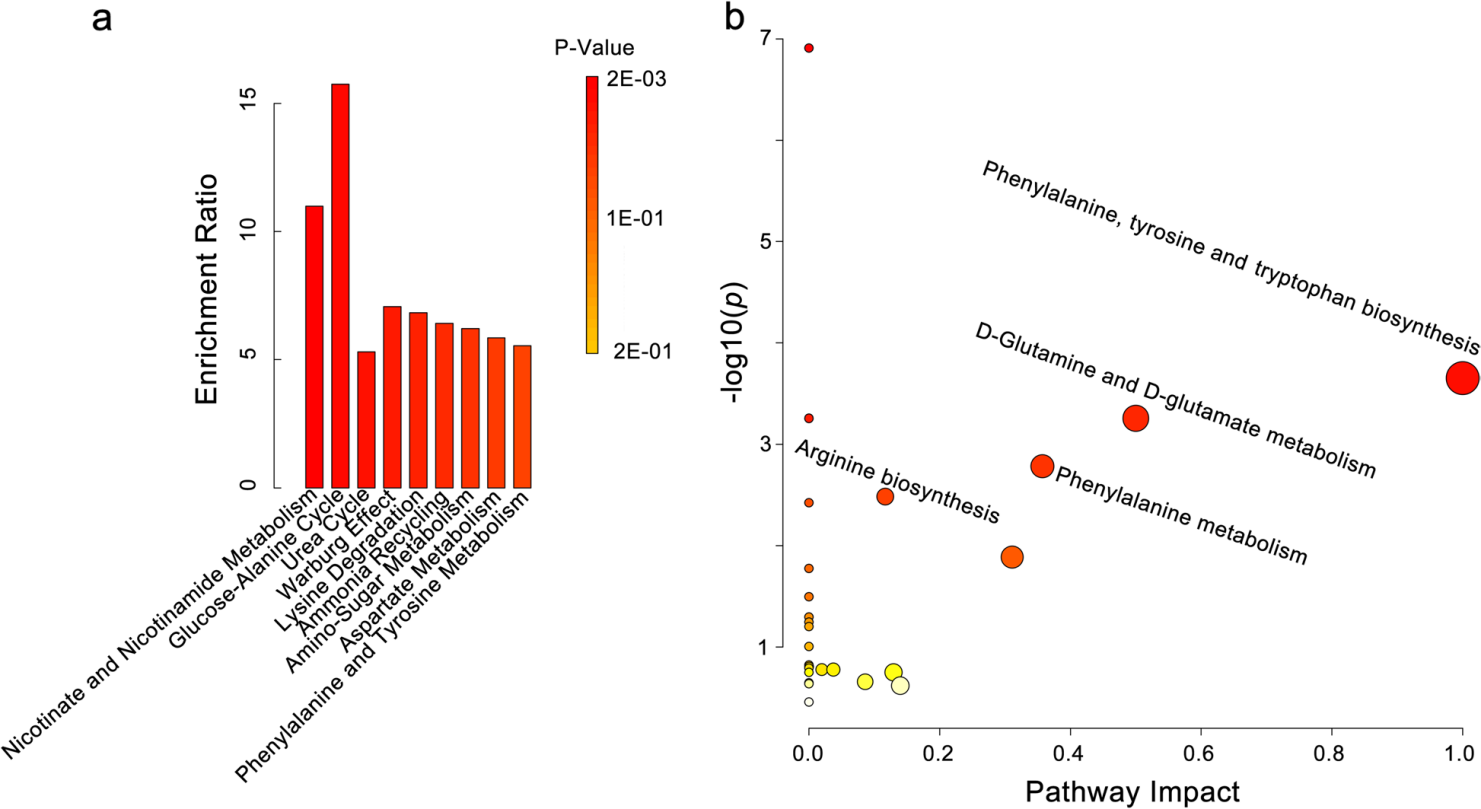
Metabolite Set Enrichment Analysis (**a**) and Pathway analysis (**b**) of the metabolites with altered abundances in cord blood of pregnant women with anti-TPO antibodies positivity. Metabolite Set Enrichment Analysis was performed by the enrichment analysis module implemented in the MetaboAnalyst web portal against the SMPDB metabolite set library. Only metabolite sets containing at least 2 entries were included. The horizontal axis denotes the enriched metabolite sets. The vertical axis denotes the enrichment ratio, computing by Hits /Expected (detailed in Table S4). The columns were colored according to the *p*-value as shown in the color bar. Pathway analysis was performed by the pathway analysis module implemented in the MetaboAnalyst web portal against the KEGG library. Hypergeometric test was used as the enrichment method, and relative-betweenness centrality was used for pathway topology analysis. Only pathway with impact > 0 and FDR-adjusted *p* < 0.05 were labeled. The corresponding data is listed in Table S5

To infer the potential metabolic pathways underlying the altered metabolites associated with anti-thyroid antibodies positivity, we performed metabolite set enrichment analysis (MESA) and metabolic pathway analysis with the implemented modules in MetaboAnalyst web portal ( https://www.metaboanalyst.ca). MESA is based on several libraries containing ~ 9, 000 biologically meaningful metabolite sets collected primarily from human studies [29]. Nine metabolite sets were enriched by MESA of the 10 altered metabolites (*P* < 0.05, Fig. 4a, Table S4), including 5 (55.56%) amino acid metabolism related, 3 (33.33%) amino residue metabolism related, 2 (22.2%) glucose metabolism related, and 1 (11.11%) vitamin metabolism metabolite sets (Nicotinate and Nicotinamide Metabolism). Among the 5 amino acid metabolism related metabolite sets, phenylalanine and tyrosine metabolism, lysine degradation, and aspartate metabolism are about amino acid degradation, while glucose-alanine cycle is about conversion of amino acid to glucose, ammonia recycling is about recycling of ammonia into central amino acid metabolism. Of the 3 amino residue metabolism related metabolite sets, urea cycle is about excretion of ammonia, while ammonia cycling is about reuse of ammonia, and amino sugar metabolism is about the transfer of amine. Of the two glucose metabolism related metabolite sets, glucose-alanine cycle is about synthesis of glucose, while the Warburg effect is about metabolism of glucose. These results indicated that anti-TPO antibodies positivity associated with synthesis and metabolism of glucose; metabolism of phenylalanine, tyrosine, lysine, and aspartate; and the excretion and recycling of ammonia.

To further investigate the disturbed metabolic pathways associated with anti-thyroid antibodies positivity, we performed Metabolic Pathway Analysis, which integrated pathway enrichment analysis and pathway topology analysis based on > 1600 pathways of 26 model organisms [29]. Seven metabolic pathways were significantly associated with anti-thyroid antibodies positivity (*FDR-adjusted p* < 0.05, Fig. 4b, Table S5), including phenylalanine, tyrosine and tryptophan biosynthesis, D-Glutamine and D-glutamate metabolism, phenylalanine metabolism, and arginine biosynthesis. From comparison of the results of MESA and pathway analysis, we proposed that anti-TPO antibodies positivity was associated with metabolic disorders in amino acid metabolism, especially in phenylalanine metabolism.

## Discussion

Anti-TPO antibodies positivity contributes to GH, which is evidenced by its correlation with higher TSH levels and higher risk of subclinical hypothyroidism [8, 30], and by the fact that women with anti-TPO antibodies positivity before pregnancy are prone to develop GH [9]. However, little is known about the intermediates between Anti-TPO antibodies positivity and GH. In the present study, we performed 1H-NMR based metabolomics on cord blood from a nested case–control cohort of pregnant women to investigate the Anti-TPO antibodies positivity related metabolic changes, in order to bring insight into the connections between Anti-TPO antibodies positivity and GH.

Anti-TPO antibodies positivity was reported to have a higher prevalence in population with obese [31] and type 1 diabetes mellitus [32], and autoimmune thyroid dysfunction was associated with serum metabolomic changes, suggesting possible correlations between anti-TPO antibodies positivity and metabolic disorder. A panel of 10 anti-TPO antibodies positivity related cord blood metabolites were identified in the present study, including 5 up-regulated and 5 down-regulated metabolites (Fig. 3, Table 2). Among the 5 up-regulated metabolites associated with anti-TPO antibodies positivity (including myo-inositol, glucose, L-glutamine, 3-hydroxybutyric acid and creatine), Myo-inositol was reported to be effective in maintaining the values of thyroid hormones (thus preventing subclinical hypothyroidism) [33] and in promoting iodine availability in thyrocytes (thus improving thyroid functionality) [34]. Maternal hyperglycemia was believed to be a risk factor for the development of thyroid autoimmunity [35]. While few connections between L-Glutamine and GH was reported, oral supplementation of glutamine was reported to reduce obesity and improve insulin sensitivity [36]. 3-hydroxybutyrate (the salt form of 3-hydroxybutyric acid) was reported to be increased in cats with overactive thyroid gland [37], and to act as a signal molecule regulating lipid metabolism and overall metabolic rate [38]. An elevated level of serum creatinine was observed in severe hypothyroidism [39]. Among the 5 down-regulated metabolites by anti-TPO antibodies positivity (including leucine, lysine, L-glutamic acid, L-tyrosine, and phenylalanine), Leucine was significantly decreased by hypothyroidism [40] and leucine supplementation was reported to improve effort tolerance of rat with hyperthyroidism [41]. Lysine deficiency contributes to disorder in plasma thyroid hormone through an elevation of FT3 [42]. Experimental hypothyroidism in rats resulted in decreased L-Glutamic acid [43], and Glutamic acid enhance thyroid stimulating hormone β subunit mRNA expression in the rat pars tuberalis [44]. Supplementation with L-Tyrosine is commonly used to support thyroid function, leading to a reduction in TSH [45, 46]. A low phenylalanine diet was report to contribute to subclinical iodine deficiency [47]. The fluctuations of all the ten metabolites induced by anti-TPO antibodies positivity in the present study were in accordance to their previously reported status in hypothyroidism, indicating that anti-TPO antibodies positivity contributes to a metabolic disorder prone to hypothyroidism.

Among the metabolic pathways associated with anti-TPO antibodies positivity observed in the present study, amino acid metabolism occupied a dominant position (Fig. 4). Accordingly, amino acid metabolism pathways were reported to be potential drug targets in autoimmune diseases [48], and played an important role in immune responses by regulating T/B lymphocytes, cellular redox state, and antibodies production [49]. Several metabolic pathways observed in the present study were reported to be significantly altered in hypothyroidism, including the down-regulation of nicotinate and nicotinamide metabolism in hypothyroidism rats [50], the increased activity of urea cycle in animal models of hypothyroidism [51], the significantly altered phenylalanine, tyrosine and tryptophan biosynthesis in GH [28], the significantly increased phenylalanine metabolism in a hypothyroidism model [52]. While increased activity of the glucose-alanine cycle was beneficial in the control of glucose utilization without increasing the risk of hypoglycemia in hyperthyroidism [53], thyroid hormone enhanced the Warburg Effect in breast cancer [54]. Phenylalanine and tyrosine metabolism was associated with serum levels of TSH and FT4 [55], and was believed to be affected by TSH [56]. The above reports supported a conclusion that the anti-TPO antibodies associated amino acid metabolism pathways were correlated with hypothyroidism or thyroid hormones.

Besides the amino acid metabolism pathways associated with anti-TPO antibodies positivity, most of the other pathways (Fig. 4) were also related to amino acid. For example, ammonia recycling is responsible for recycling of ammonia into central amino acid metabolism [57], Urea Cycle converts excess ammonia into urea in the mitochondria of liver cells [58], Glucose-alanine Cycle refers to the series of reactions in which amino groups and carbons from muscle are transported to the liver [59]. From the results of MESA (Fig. 4a) and pathway analysis (Fig. 4b), we concluded that the anti-TPO antibodies positivity associated metabolic disorder contributed to hypothyroidism possibly through disturbance of amino acid metabolism in cord blood of pregnant women.

As a shortcoming of the present study, the number of enrolled cases is relatively small; this may result in false negative or false positive results due to the complicated physiological status among pregnant women. The fetal metabolic disorder associated with anti-TPO antibodies positivity may be affected by other clinical factors besides those included in the present study. Further large-scale studies are recommended to further explore the roles of anti-TPO antibodies positivity associated cord blood metabolic disorder observed in this study.

In summary, we performed 1H-NMR metabolomics on cord blood of a nested case–control cohort of 22 pregnant women to reveal the anti-TPO antibodies positivity associated fetal metabolic disorder. A panel of 10 metabolites with altered abundances was observed, all of which have been reported to be associated with hypothyroidism. The results of the present study suggested a correlation between anti-TPO antibodies positivity and fetal metabolic disorder, and called more attention to the clinical outcome of gestational anti-TPO antibodies positivity to both postpartum and the newborn health.

## Supplementary Information

**Additional file 1:** **Figure S1. **Effects of normalization to the 1H-NMRmetabolomic profiling data.The normalization procedures implemented in theMetaboAnalyst web portal (https://www.metaboanalyst.ca) was applied for datanormalization, including sample normalization (normalized by sum), datatransformation (log transformation), and data scaling (mean-centered anddivided by the square root of the standard deviation of each variable). Theleft panels represent the metabolomic data before normalization, and the rightpanels represent the metabolomic data after normalization.

**Additional file 2:** **Figure S2.** Quality control of the metabolomic profiling based on PCA score plot. PCA was performed on the 1H-NMR metabolomic profiling data of the clinical samples and the quality controls. The blue solid circles denote the anti-TPO antibodies positivity group, the green solid circles denote the nest control group, the red solid circles denote the QCs. The ellipse represents the 95% confidence interval using Hotelling’s T^2^ statistics.

**Additional file 3:** **Figure S3.** Hotelling’s T2 plot (a) and permutationtest plot of the OPLS-DA model (b). (a) Hotelling’s T2 plot of the OPLS-DA model (Figure1b) was generated by SIMCA-P. The x-axis denotes the 1H-NMR features, they-axis denotes the T2 Range. The red dash line represents the 99% critical limitof T2, the yellow dash line represents the 95% critical limit of T2. (b) Plotof R2Y and Q2 from a 200-step permutation test to the OPLS-DA model. The y-axisshows the value of R2Y and Q2, the x-axis shows the correlation coefficient betweenthe observed and the permuted data. The two points on the upper-right representthe R2Y and Q2 from the observed data set as labeled. The other points on thebottom-left correspond to R2Y and Q2 from the permuted data sets.

**Additional file 4:** **Table S1. **The 1H-NMRfeatures with VIP > 1 corresponding to Fig. 2b.** Table S2. **The 1H-NMRfeatures with absolute p(corr) value > 0.40 corresponding to Fig. 2c. **Table S3. **Datacorresponding to the volcano plot in Fig. 2d. **Table S4. **Metaboliteset enrichment analysis results corresponding to Fig. 4a. **Table S5. **The results ofmetabolic pathway analysis corresponding to Fig. 4b.

## Data Availability

The dataset supporting the conclusions of this article is included within the article.

## Abbreviations

1H-NMR: Hydrogen-1 Nuclear Magnetic Resonance
TSP: 3-Trimethylsilyl-[2,2,3,3-D4]-Propionate
HMDB: Human Metabolome Database
VIP: Variable Importance for the Projection
FDR: False Discovery Rate
PCA: Principal Component Analysis
OPLS-DA: Orthogonal Projection to Latent Structures Discriminant Analysis
anti-TPO: Anti-Thyroid Peroxidase
GH: Gestational Hypothyroidism
BMI: Body Mass Index
TSH: Thyroid Stimulating Hormone
FT3: Free Triiodothyronine
FT4: Free Thyroxine
