# Supplementary figures and images for: Cord blood metabolomics reveals gestational metabolic disorder associated with anti-thyroid peroxidase antibodies positivity
Source: BMC Pregnancy Childbirth. 2022 Mar 24;22:244. doi: 10.1186/s12884-022-04564-8 (PMC8952885; doi:10.1186/s12884-022-04564-8)

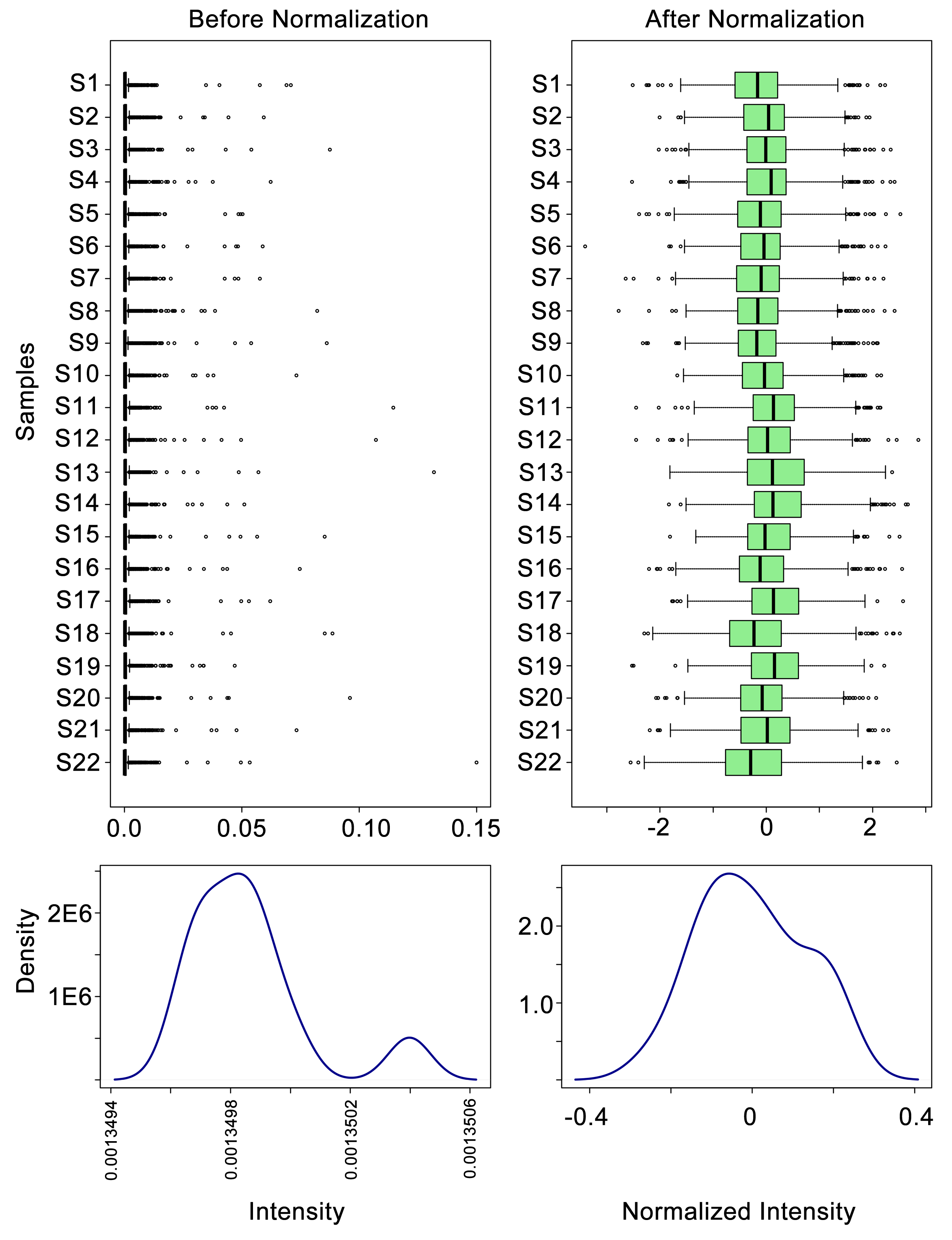

Supplement: Supplementary file 1 — Additional file 1: Figure S1. Effects of normalization to the 1H-NMRmetabolomic profiling data.The normalization procedures implemented in theMetaboAnalyst web portal (https://www.metaboanalyst.ca) was applied for datanormalization, including sample normalization (normalized by sum), datatransformation (log transformation), and data scaling (mean-centered anddivided by the square root of the standard deviation of each variable). Theleft panels represent the metabolomic data before normalization, and the rightpanels represent the metabolomic data after normalization. [file 12884_2022_4564_MOESM1_ESM.tif]

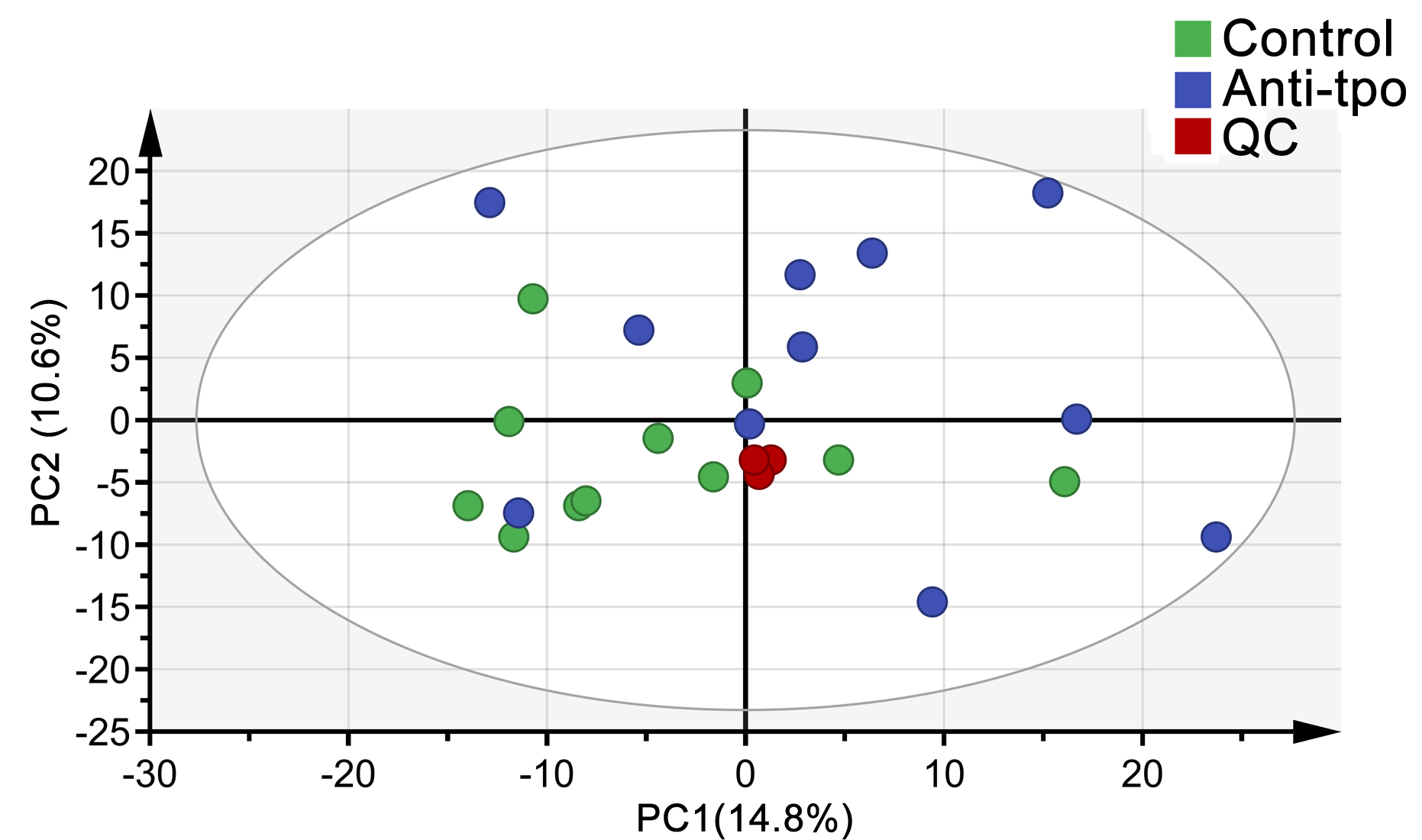

Supplement: Supplementary file 2 — Additional file 2: Figure S2. Quality control of the metabolomic profiling based on PCA score plot. PCA was performed on the 1H-NMR metabolomic profiling data of the clinical samples and the quality controls. The blue solid circles denote the anti-TPO antibodies positivity group, the green solid circles denote the nest control group, the red solid circles denote the QCs. The ellipse represents the 95% confidence interval using Hotelling’s T2 statistics. [file 12884_2022_4564_MOESM2_ESM.tif]

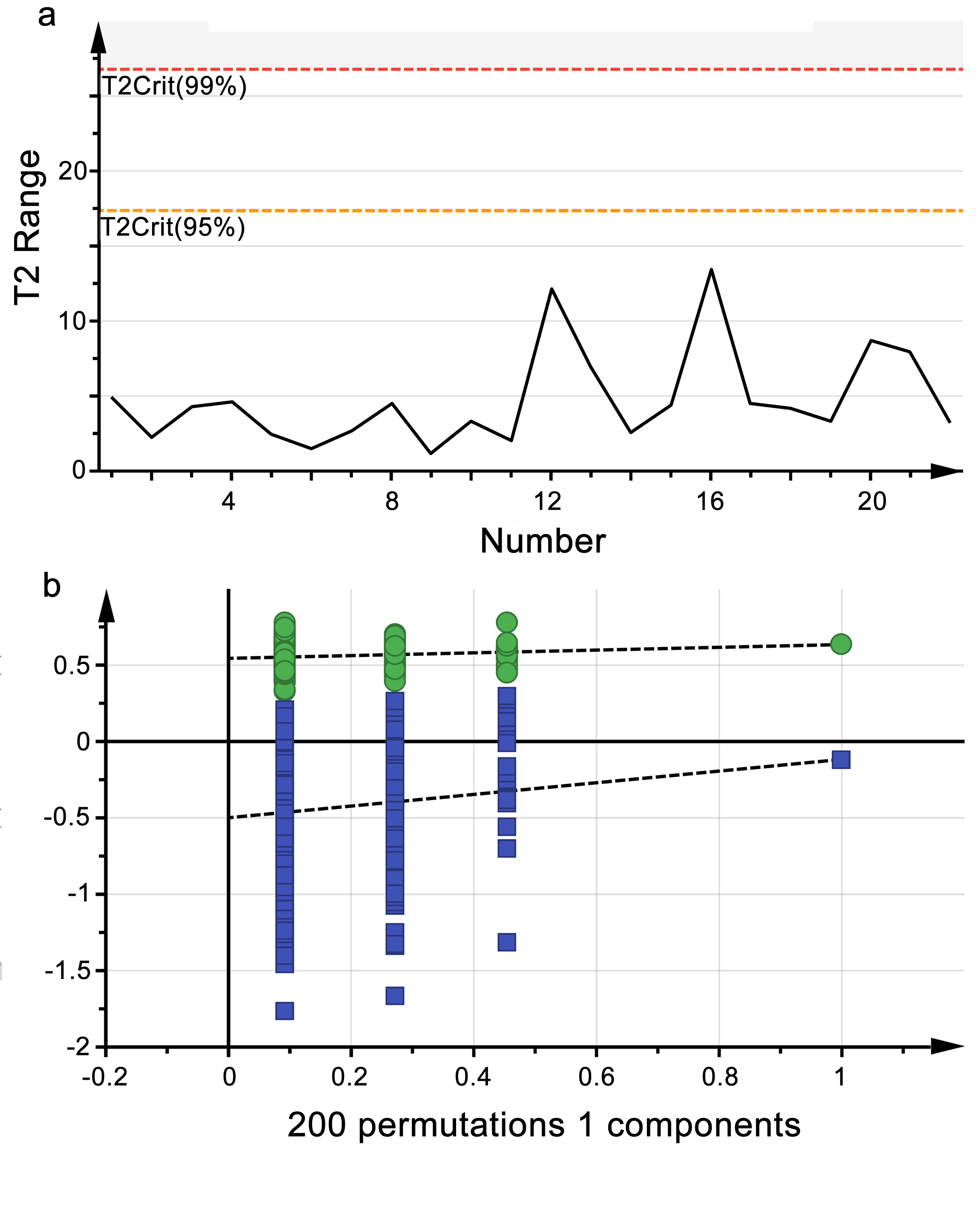

Supplement: Supplementary file 3 — Additional file 3: Figure S3. Hotelling’s T2 plot (a) and permutationtest plot of the OPLS-DA model (b). (a) Hotelling’s T2 plot of the OPLS-DA model (Figure1b) was generated by SIMCA-P. The x-axis denotes the 1H-NMR features, they-axis denotes the T2 Range. The red dash line represents the 99% critical limitof T2, the yellow dash line represents the 95% critical limit of T2. (b) Plotof R2Y and Q2 from a 200-step permutation test to the OPLS-DA model. The y-axisshows the value of R2Y and Q2, the x-axis shows the correlation coefficient betweenthe observed and the permuted data. The two points on the upper-right representthe R2Y and Q2 from the observed data set as labeled. The other points on thebottom-left correspond to R2Y and Q2 from the permuted data sets. [file 12884_2022_4564_MOESM3_ESM.tif]
