# Supplementary material for: Cord blood metabolomics reveals gestational metabolic disorder associated with anti-thyroid peroxidase antibodies positivity
Source: BMC Pregnancy Childbirth. 2022 Mar 24;22:244. doi: 10.1186/s12884-022-04564-8 (PMC8952885; doi:10.1186/s12884-022-04564-8)
Supplement: Supplementary file 4 — Additional file 4: Table S1. The 1H-NMRfeatures with VIP > 1 corresponding to Fig. 2b. Table S2. The 1H-NMRfeatures with absolute p(corr) value > 0.40 corresponding to Fig. 2c. Table S3. Datacorresponding to the volcano plot in Fig. 2d. Table S4. Metaboliteset enrichment analysis results corresponding to Fig. 4a. Table S5. The results ofmetabolic pathway analysis corresponding to Fig. 4b. [file 12884_2022_4564_MOESM4_ESM.doc]

| **Table S1. The 1H-NMR features with VIP > 1 corresponding to Figure 2b.** | | | | | |
| --- | --- | --- | --- | --- | --- |
| **1H-NMR  Feature** | **VIP value** | **1H-NMR  Feature** | **VIP value** | **1H-NMR  Feature** | **VIP value** |
| 0.02 | 2.25 | 1.99 | 1.86 | 3.77 | 1.50 |
| 0.03 | 1.65 | 2.00 | 2.10 | 3.78 | 2.20 |
| 0.04 | 1.11 | 2.01 | 2.09 | 3.80 | 2.48 |
| 0.05 | 1.08 | 2.02 | 2.05 | 3.81 | 1.31 |
| 0.83 | 1.68 | 2.03 | 1.75 | 3.84 | 1.65 |
| 0.84 | 2.82 | 2.04 | 1.30 | 3.85 | 1.33 |
| 0.85 | 3.88 | 2.06 | 2.55 | 3.86 | 3.11 |
| 0.86 | 3.78 | 2.07 | 1.20 | 3.88 | 1.71 |
| 0.87 | 2.83 | 2.25 | 1.31 | 3.91 | 3.73 |
| 0.88 | 1.96 | 2.40 | 1.32 | 3.93 | 2.83 |
| 0.89 | 1.17 | 2.41 | 1.05 | 4.06 | 1.09 |
| 0.94 | 1.06 | 2.43 | 1.00 | 4.08 | 1.17 |
| 0.95 | 1.22 | 2.45 | 1.99 | 4.11 | 1.07 |
| 0.96 | 1.45 | 2.47 | 1.11 | 4.12 | 2.86 |
| 0.97 | 1.81 | 3.04 | 1.28 | 4.13 | 3.63 |
| 0.98 | 1.83 | 3.05 | 1.17 | 4.14 | 3.06 |
| 1.00 | 1.61 | 3.25 | 5.86 | 4.15 | 2.94 |
| 1.02 | 1.91 | 3.26 | 2.83 | 4.16 | 3.44 |
| 1.05 | 1.16 | 3.27 | 2.50 | 4.17 | 1.42 |
| 1.07 | 1.87 | 3.28 | 1.13 | 4.18 | 1.09 |
| 1.23 | 1.08 | 3.29 | 2.38 | 4.19 | 1.08 |
| 1.24 | 3.23 | 3.30 | 1.80 | 4.32 | 1.02 |
| 1.25 | 3.47 | 3.31 | 1.35 | 4.33 | 1.07 |
| 1.26 | 3.61 | 3.32 | 1.31 | 4.34 | 1.14 |
| 1.27 | 3.36 | 3.34 | 1.14 | 4.58 | 1.05 |
| 1.28 | 3.07 | 3.41 | 1.60 | 4.59 | 1.28 |
| 1.29 | 2.90 | 3.43 | 2.98 | 4.60 | 1.25 |
| 1.30 | 1.77 | 3.44 | 3.26 | 4.64 | 1.12 |
| 1.31 | 1.23 | 3.47 | 2.92 | 4.65 | 1.45 |
| 1.35 | 4.33 | 3.48 | 2.72 | 4.66 | 4.77 |
| 1.36 | 8.19 | 3.50 | 2.97 | 4.67 | 5.00 |
| 1.37 | 1.10 | 3.51 | 1.02 | 5.25 | 1.26 |
| 1.49 | 1.81 | 3.54 | 1.84 | 5.26 | 1.12 |
| 1.51 | 1.11 | 3.55 | 1.36 | 5.27 | 1.01 |
| 1.72 | 1.01 | 3.57 | 1.29 | 5.34 | 1.01 |
| 1.76 | 1.03 | 3.72 | 1.55 | 5.81 | 1.15 |
| 1.92 | 1.05 | 3.74 | 3.22 | 5.82 | 1.17 |
| 1.97 | 1.11 | 3.75 | 1.42 | 5.83 | 1.02 |
| 1.98 | 1.42 | 3.76 | 2.95 | 7.82 | 1.13 |
|  |  |  |  | 7.84 | 1.06 |

| **Table S2. The 1H-NMR features with absolute p(corr) value > 0.40 corresponding to Figure 2c.** | | | | | | | | |
| --- | --- | --- | --- | --- | --- | --- | --- | --- |
| **1H-NMR  feature ID** | **p[1]** | **p(corr)[1]** | **1H-NMR  feature ID** | **p[1]** | **p(corr)[1]** | **1H-NMR  feature ID** | **p[1]** | **p(corr)[1]** |
| 0.03 | -0.06 | -0.45 | 2.21 | 0.03 | 0.66 | 4.65 | 0.06 | 0.43 |
| 0.05 | -0.04 | -0.45 | 2.22 | 0.03 | 0.63 | 4.66 | 0.18 | 0.58 |
| 0.06 | -0.04 | -0.46 | 2.37 | 0.04 | 0.44 | 4.67 | 0.19 | 0.81 |
| 0.07 | -0.04 | -0.47 | 2.40 | 0.05 | 0.52 | 5.36 | 0.03 | 0.49 |
| 0.48 | -0.01 | -0.47 | 2.45 | 0.08 | 0.76 | 5.37 | 0.02 | 0.52 |
| 0.59 | -0.01 | -0.44 | 2.50 | 0.03 | 0.44 | 5.38 | 0.02 | 0.58 |
| 0.65 | -0.01 | -0.43 | 2.65 | 0.03 | 0.45 | 5.39 | 0.01 | 0.51 |
| 0.66 | 0.01 | 0.48 | 2.88 | 0.03 | 0.43 | 5.50 | 0.01 | 0.44 |
| 0.67 | 0.01 | 0.44 | 2.90 | 0.03 | 0.47 | 5.61 | -0.01 | -0.57 |
| 0.77 | 0.02 | 0.43 | 3.04 | -0.05 | -0.44 | 5.71 | 0.01 | 0.44 |
| 0.78 | 0.02 | 0.48 | 3.29 | 0.09 | 0.49 | 5.79 | 0.03 | 0.44 |
| 0.83 | -0.07 | -0.59 | 3.30 | 0.07 | 0.63 | 5.80 | 0.04 | 0.52 |
| 0.84 | -0.11 | -0.66 | 3.31 | 0.05 | 0.59 | 5.81 | 0.04 | 0.57 |
| 0.85 | -0.15 | -0.74 | 3.32 | 0.05 | 0.57 | 5.82 | 0.05 | 0.62 |
| 0.86 | -0.15 | -0.65 | 3.34 | 0.04 | 0.60 | 5.83 | 0.04 | 0.61 |
| 0.87 | -0.11 | -0.45 | 3.47 | -0.11 | -0.53 | 5.84 | 0.03 | 0.55 |
| 0.95 | -0.05 | -0.47 | 3.48 | -0.11 | -0.44 | 5.85 | 0.03 | 0.56 |
| 0.96 | -0.06 | -0.57 | 3.54 | 0.07 | 0.66 | 5.86 | 0.02 | 0.52 |
| 0.97 | -0.07 | -0.67 | 3.74 | -0.12 | -0.44 | 5.87 | 0.02 | 0.52 |
| 0.98 | -0.07 | -0.63 | 3.76 | -0.11 | -0.49 | 5.89 | 0.01 | 0.45 |
| 1.00 | -0.06 | -0.43 | 3.78 | -0.09 | -0.50 | 5.95 | 0.01 | 0.55 |
| 1.02 | -0.07 | -0.51 | 3.80 | -0.10 | -0.51 | 6.27 | 0.01 | 0.50 |
| 1.07 | -0.07 | -0.57 | 3.86 | -0.12 | -0.44 | 6.45 | 0.01 | 0.45 |
| 1.09 | 0.02 | 0.44 | 3.91 | -0.14 | -0.50 | 6.72 | 0.01 | 0.42 |
| 1.11 | 0.03 | 0.65 | 3.93 | -0.11 | -0.46 | 6.83 | -0.02 | -0.53 |
| 1.12 | 0.02 | 0.58 | 3.98 | 0.03 | 0.45 | 6.84 | -0.02 | -0.56 |
| 1.24 | -0.13 | -0.73 | 4.06 | 0.04 | 0.59 | 6.86 | 0.01 | 0.45 |
| 1.25 | -0.13 | -0.74 | 4.08 | 0.05 | 0.53 | 6.93 | -0.03 | -0.49 |
| 1.26 | -0.14 | -0.70 | 4.11 | 0.04 | 0.54 | 6.96 | -0.01 | -0.57 |
| 1.27 | -0.13 | -0.60 | 4.12 | 0.11 | 0.67 | 6.99 | -0.02 | -0.61 |
| 1.28 | -0.12 | -0.48 | 4.13 | 0.14 | 0.52 | 7.02 | 0.01 | 0.49 |
| 1.36 | 0.32 | 0.50 | 4.14 | 0.12 | 0.44 | 7.09 | -0.03 | -0.43 |
| 1.70 | -0.02 | -0.43 | 4.15 | 0.11 | 0.64 | 7.10 | 0.02 | 0.49 |
| 1.71 | -0.02 | -0.44 | 4.16 | 0.13 | 0.81 | 7.14 | 0.01 | 0.56 |
| 1.72 | -0.04 | -0.64 | 4.17 | 0.06 | 0.49 | 7.21 | -0.03 | -0.54 |
| 1.73 | -0.03 | -0.44 | 4.19 | 0.04 | 0.50 | 7.29 | 0.01 | 0.46 |
| 1.74 | -0.03 | -0.44 | 4.20 | 0.04 | 0.57 | 7.37 | 0.01 | 0.49 |
| 1.76 | -0.04 | -0.54 | 4.21 | 0.03 | 0.43 | 7.43 | 0.02 | 0.42 |
| 1.83 | 0.01 | 0.45 | 4.29 | 0.04 | 0.43 | 7.56 | 0.01 | 0.44 |
| 1.85 | 0.02 | 0.56 | 4.31 | 0.04 | 0.45 | 7.81 | -0.02 | -0.43 |
| 1.91 | -0.04 | -0.46 | 4.32 | 0.04 | 0.46 | 7.82 | -0.04 | -0.44 |
| 1.92 | -0.04 | -0.45 | 4.33 | 0.04 | 0.49 | 7.84 | 0.04 | 0.49 |
| 1.95 | -0.04 | -0.52 | 4.34 | 0.04 | 0.51 | 7.85 | 0.02 | 0.55 |
| 1.96 | -0.04 | -0.53 | 4.35 | 0.04 | 0.44 | 7.92 | -0.01 | -0.42 |
| 1.97 | -0.04 | -0.62 | 4.36 | 0.03 | 0.43 | 7.94 | 0.01 | 0.53 |
| 1.98 | -0.06 | -0.72 | 4.37 | 0.04 | 0.49 | 7.95 | 0.01 | 0.42 |
| 1.99 | -0.07 | -0.82 | 4.38 | 0.03 | 0.42 | 7.96 | 0.01 | 0.59 |
| 2.00 | -0.08 | -0.80 | 4.39 | 0.03 | 0.44 | 8.00 | -0.01 | -0.52 |
| 2.01 | -0.08 | -0.70 | 4.40 | 0.03 | 0.48 | 8.03 | 0.01 | 0.45 |
| 2.02 | -0.08 | -0.63 | 4.46 | 0.03 | 0.43 | 8.18 | -0.01 | -0.56 |
| 2.03 | -0.07 | -0.53 | 4.56 | 0.03 | 0.48 | 8.43 | -0.01 | -0.43 |
| 2.04 | -0.05 | -0.48 | 4.57 | 0.03 | 0.54 | 8.47 | 0.01 | 0.45 |
| 2.06 | -0.10 | -0.59 | 4.58 | 0.04 | 0.54 |  |  |  |
| 2.09 | -0.03 | -0.45 | 4.59 | 0.05 | 0.62 |  |  |  |
| 2.20 | 0.03 | 0.56 | 4.60 | 0.05 | 0.61 |  |  |  |

| **Table S3. Data corresponding to the volcano plot in Figure 2d.** | | | | |
| --- | --- | --- | --- | --- |
| **1H-NMR**  **feature ID** | **Identified Metabolites** | **Fold Change** | **log2(FC)** | **FDR-adjusted**  ***p*-value** |
| 0.28 |  | 0.04 | -4.64 | 4.62E-02 |
| 1.72 | L-Leucine | 0.06 | -4.00 | 8.72E-03 |
| 1.95 | L-Lysine | 0.18 | -2.48 | 1.21E-02 |
| 1.99 | L-Glutamic acid | 0.14 | -2.84 | 3.11E-02 |
| 2.21 |  | 0.45 | -1.15 | 1.39E-02 |
| 2.45 | L-Glutamine | 4.73 | 2.24 | 1.56E-02 |
| 3.54 |  | 2.05 | 1.04 | 3.94E-02 |
| 4.06 | Creatinine | 17.67 | 4.14 | 1.47E-02 |
| 4.08 | myo-inositol | 4.61 | 2.21 | 4.10E-03 |
| 4.16 | 3-Hydroxybutyric acid | 2.06 | 1.04 | 2.36E-02 |
| 4.17 |  | 2.09 | 1.06 | 9.33E-03 |
| 4.20 |  | 2.09 | 1.06 | 1.79E-02 |
| 4.23 |  | 2.10 | 1.07 | 3.96E-02 |
| 4.24 |  | 2.14 | 1.10 | 4.66E-02 |
| 4.38 |  | 21.16 | 4.40 | 5.75E-03 |
| 4.44 |  | 2.47 | 1.30 | 2.53E-02 |
| 4.45 |  | 2.90 | 1.54 | 5.49E-04 |
| 4.46 |  | 2.91 | 1.54 | 2.24E-02 |
| 4.56 |  | 2.95 | 1.56 | 1.27E-02 |
| 4.59 |  | 4.26 | 2.09 | 3.90E-02 |
| 4.60 |  | 4.31 | 2.11 | 1.51E-02 |
| 4.67 | D-Glucose | 2.10 | 1.07 | 3.95E-03 |
| 5.51 |  | 4.76 | 2.25 | 1.85E-02 |
| 5.53 |  | 4.94 | 2.31 | 4.28E-04 |
| 5.70 |  | 5.37 | 2.42 | 2.38E-02 |
| 5.83 |  | 5.61 | 2.49 | 4.95E-02 |
| 5.84 |  | 5.82 | 2.54 | 1.07E-02 |
| 5.85 |  | 6.26 | 2.65 | 3.41E-02 |
| 5.93 |  | 6.55 | 2.71 | 4.62E-02 |
| 6.40 |  | 6.99 | 2.81 | 2.38E-03 |
| 6.72 |  | 7.33 | 2.87 | 2.63E-02 |
| 6.86 |  | 9.35 | 3.23 | 3.00E-02 |
| 6.96 |  | 9.55 | 3.26 | 1.53E-02 |
| 7.21 | L-Tyrosine | 0.34 | -1.54 | 2.04E-02 |
| 7.36 | Phenylalanine | 0.16 | -2.68 | 3.00E-02 |
| 7.37 |  | 9.71 | 3.28 | 4.16E-03 |
| 7.48 |  | 11.86 | 3.57 | 2.32E-02 |
| 8.00 |  | 12.21 | 3.61 | 3.64E-02 |
| 8.23 |  | 21.41 | 4.42 | 6.04E-03 |
| 8.38 |  | 36.63 | 5.19 | 5.24E-03 |
| 8.43 |  | 89.19 | 6.48 | 4.07E-02 |

| **Table S4. Metabolite set enrichment analysis results corresponding to Figure 4a.** | | | | |  |
| --- | --- | --- | --- | --- | --- |
| **Metabolite Set** | **Total** | **Hits** | **Expect** | ***P-*Value** | |
| Phenylalanine and Tyrosine Metabolism | 28 | 3 | 0.27 | 1.94E-03 | |
| Glucose-Alanine Cycle | 13 | 2 | 0.13 | 6.33E-03 | |
| Warburg Effect | 58 | 3 | 0.57 | 1.56E-02 | |
| Urea Cycle | 29 | 2 | 0.28 | 3.03E-02 | |
| Lysine Degradation | 30 | 2 | 0.29 | 3.23E-02 | |
| Ammonia Recycling | 32 | 2 | 0.31 | 3.64E-02 | |
| Amino Sugar Metabolism | 33 | 2 | 0.32 | 3.86E-02 | |
| Aspartate Metabolism | 35 | 2 | 0.34 | 4.30E-02 | |
| Nicotinate and Nicotinamide Metabolism | 37 | 2 | 0.36 | 4.76E-02 | |

| **Table S5. The results of metabolic pathway analysis corresponding to Figure 4b.** | | | | |  |  |
| --- | --- | --- | --- | --- | --- | --- |
| **Pathway Name** | **Match Status** | **p** | **-log(p)** | **Holm P** | **FDR-adjusted**  ***p*-value** | **Impact** |
| Aminoacyl-tRNA biosynthesis | 6/48 | 1.23E-07 | 6.91 | 1.03E-05 | 1.03E-05 | 0.00 |
| Phenylalanine, tyrosine and tryptophan biosynthesis | 2/4 | 2.23E-04 | 3.65 | 1.85E-02 | 9.38E-03 | 1.00 |
| Nitrogen Metabolism | 2/6 | 5.55E-04 | 3.26 | 4.55E-02 | 1.16E-02 | 0.00 |
| D-Glutamine and D-glutamate metabolism | 2/6 | 5.55E-04 | 3.26 | 4.55E-02 | 1.16E-02 | 0.50 |
| Phenylalanine metabolism | 2/10 | 1.64E-03 | 2.78 | 1.31E-01 | 2.76E-02 | 0.36 |
| Arginine biosynthesis | 2/14 | 3.27E-03 | 2.49 | 2.59E-01 | 4.52E-02 | 0.12 |
| Butanoate metabolism | 2/15 | 3.76E-03 | 2.42 | 2.94E-01 | 4.52E-02 | 0.00 |
| Alanine, aspartate and glutamate metabolism | 2/28 | 1.30E-02 | 1.89 | 9.97E-01 | 1.40E-01 | 0.31 |
| Glyoxylate and dicarboxylate metabolism | 2/32 | 1.68E-02 | 1.78 | 1.00 | 1.56E-01 | 0.00 |
| Synthesis and degradation of ketone bodies | 1/5 | 3.19E-02 | 1.50 | 1.00 | 2.68E-01 | 0.00 |

**Supplementary figure legends**

**Figure S1. Effects of normalization to the 1H-NMR metabolomic profiling data.** The normalization procedures implemented in the MetaboAnalyst web portal (https://www.metaboanalyst.ca) was applied for data normalization, including sample normalization (normalized by sum), data transformation (log transformation), and data scaling (mean-centered and divided by the square root of the standard deviation of each variable). The left panels represent the metabolomic data before normalization, and the right panels represent the metabolomic data after normalization.

**Figure S2. Quality control of the metabolomic profiling based on PCA score plot.** PCA was performed on the 1H-NMR metabolomic profiling data of the clinical samples and the quality controls. The blue solid circles denote the anti-TPO antibodies positivity group, the green solid circles denote the nest control group, the red solid circles denote the QCs. The ellipse represents the 95% confidence interval using Hotelling’s T^2^ statistics.

**Figure S3. Hotelling’s T2 plot (a) and permutation test plot of the OPLS-DA model (b).** (a) Hotelling’s T2 plot of the OPLS-DA model (Figure 1b) was generated by SIMCA-P. The x-axis denotes the 1H-NMR features, the y-axis denotes the T2 Range. The red dash line represents the 99% critical limit of T2, the yellow dash line represents the 95% critical limit of T2. (b) Plot of R2Y and Q2 from a 200-step permutation test to the OPLS-DA model. The y-axis shows the value of R2Y and Q2, the x-axis shows the correlation coefficient between the observed and the permuted data. The two points on the upper-right represent the R2Y and Q2 from the observed data set as labeled. The other points on the bottom-left correspond to R2Y and Q2 from the permuted data sets.
